# A Rasch analysis of the Person-Centred Climate Questionnaire – staff version

**DOI:** 10.1186/s12913-019-4803-9

**Published:** 2019-12-26

**Authors:** Mark Wilberforce, Anders Sköldunger, David Edvardsson

**Affiliations:** 1 Social Policy Research Unit, Department of Social Policy and Social Work, University of York, York, UK; 2 Personal Social Services Research Unit, University of Manchester, Manchester, UK; 3 Department of Nursing, Umeå University, Umeå, Sweden; 4 School of Nursing and Midwifery, La Trobe University, Bundoora, Australia

**Keywords:** Person-centred care, Psychometrics, Dementia, Care quality, Rasch analysis

## Abstract

**Background:**

Person-centred care is the bedrock of modern dementia services, yet the evidence-base to support its implementation is not firmly established. Research is hindered by a need for more robust measurement instruments. The 14-item Person-Centred Climate Questionnaire - Staff version (PCQ-S) is one of the most established scales and has promising measurement properties. However, its construction under classical test theory methods leaves question marks over its rigour and the need for evaluation under more modern testing procedures.

**Methods:**

The PCQ-S was self-completed by nurses and other care staff working across nursing homes in 35 Swedish municipalities in 2013/14. A Rasch analysis was undertaken in RUMM2030 using a partial credit model suited to the Likert-type items. Three subscales of the PCQ-S were evaluated against common thresholds for overall fit to the Rasch model; ordering of category thresholds; unidimensionality; local dependency; targeting; and Differential Item Functioning. Three subscales were evaluated separately as unidimensional models and then combined as subtests into a single measure. Due to large number of respondents (*n* = 4381), two random sub-samples were drawn, with a satisfactory model established in the first (‘evaluation’) and confirmed in the second (‘validation’). Final item locations and a table converting raw scores to Rasch-transformed values were created using the full sample.

**Results:**

All three subscales had disordered thresholds for some items, which were resolved by collapsing categories. The three subscales fit the assumptions of the Rasch model after the removal of two items, except for subscale 3, where there was evidence of local dependence between two items. By forming subtests, the 3 subscales were combined into a single Rasch model which had satisfactory fit statistics. The Rasch form of the instrument (PCQ-S-R) had an adequate but modest Person Separation Index (< 0.80) and some evidence of mistargeting due to a low number of ‘difficult-to-endorse’ items.

**Conclusions:**

The PCQ-S-R has 12 items and can be used as a unidimensional scale with interval level properties, using the nomogram presented within this paper. The scale is reliable but has some inefficiencies due to too few high-end thresholds inhibiting discrimination amongst populations who already perceive that person-centred care is very good in their environment.

## Background

Person-centredness is internationally regarded as an essential design principle underpinning modern dementia care services. Although tracing its roots to Rogerian psychotherapy [1], the seminal works of Tom Kitwood [2] are seen as defining the starting-point of person-centred dementia care, which seeks to address how perceptions of dementia can detrimentally affect a person’s standing in relation to those around them [3]. Gerontological nursing has since produced a healthy stock of related conceptual advances, particularly in developing enabling care relationships [4–6]. Each share social constructionist perspectives of ageing [7, 8] and are concerned with how identity, personhood and agency can be reinforced or compromised depending on the nature of the care environment [9]. Although there is no universally agreed definition, most articulations describe care based on a holistic understanding of a person’s lived experiences; providing a care environment congruent with their preferences and that encourages expression of self; promoting their place as valued members of social relationships and networks; and tailoring support to the individual [3, 5, 10].

The rise of person-centredness in dementia care finds support in an encouraging, if not definitive, evidence-base. Experimental designs have associated person-centred approaches with reduced behavioural symptoms (particularly agitation) and use of neuroleptics with care home residents [11–13]; with observational and qualitative studies also having linked person-centredness with improved wellbeing and physical health outcomes [14], and benefits for care workers [15]. However, evidence is inconsistent, and there is a dearth of research exploring the importance of *how* person-centredness is best implemented. A lack of high quality measurement instruments has been widely highlighted as one impediment to rigorous research [16, 17]. The most established measures, particularly Dementia Care Mapping [18], demands intensive recording of care interactions by specially-trained observers which is beyond the resources of many services and research groups. The need for robust questionnaire-based instruments has been highlighted [17].

### The Person-Centred Climate Questionnaire

The Person-Centred Climate Questionnaire is one of the most well-documented and widely tested scales available for evaluating the person-centred quality of the care environment within institutional settings [17, 19]. It is based on an empirically-developed theory of how supportive care environments can protect personhood in the context of cognitive decline and beyond [19], and comprises 14 statements for respondents to assess their agreement with on a 6-point Likert scale (1 = No, I disagree completely, to 6 = Yes, I agree completely). The staff version (PCQ-S) is a proxy report version for use in care settings where an expected high prevalence of cognitive impairment/ dementia would inhibit self-report responses. Its 14 items are organized into three subscales - see Table 1 – spanning safety, homeliness and community. The original PCQ-S was developed in Swedish, but empirically-tested English, Norwegian, Slovenian, Chinese and Korean versions have been published [20–23]. A patient-completed version (PCQ-P) is also available, although that is not the subject of the present article [24].

**Table 1:** Items and factors of the PCQ-S

Scale 1: A climate of safety	Scale 2: A climate of everydayness	Scale 3: A climate of community
1. A place where I feel welcome	6. A place which feels homely even though it is in an institution	11. A place where it is easy for the patients to keep in contact with their loved ones
2. A place where I feel acknowledged as a person	7. A place where there is something nice to look at	12. A place where it is easy for the patients to receive visitors
3. A place where I feel I can be myself	8. A place where it is quiet and peaceful	13. A place where it is easy for the patients to talk to the staff
4. A place where the patients are in safe hands	9. A place where it is possible to get unpleasant thoughts out of your head	14. A place where the patients have someone to talk to if they so wish.
5. A place where the staff use a language that the patients can understand	10. A place which is neat and clean	

The strengths of the PCQ-S include its encouraging measurement properties, established across an array of empirical studies. Content validity has been supported by expert agreement methods [25] whilst repeated factor analytic studies in independent populations have supported a reasonably stable three factor structure [26]. Cronbach alpha for the subscales, and for the global score, are consistently estimated above 0.80 – even in other languages it has been translated into. Specific studies of reliability and cut-scores have been undertaken, providing greater utility for application in practice [27]. The PCQ-S is firmly established as a regular research tool in psychosocial studies of dementia care and its implications for patient welfare and staff satisfaction [28–30].

However, the PCQ-S was developed under classical test theory (CTT) which has been subject to increasing criticism as the appropriate framework for measurement instruments [31]. Four common limitations of CTT are highlighted here. First, the assumption that ordinal Likert-type items can be summed to form a measure with interval-level properties remains to be proven [32]. A significant leap of faith is necessary for the score of one point on an ordinally-constructed instrument to be assumed truly equal across the entire length of the scale. If this assumption is breached, parametric tests are not supported and even simple mathematical operations (e.g. mean scores) are then inappropriate [32, 33]. Second, reliability estimates are commonly thought to be artificially inflated in the presence of locally dependent items; whereby the pursuit of high Cronbach alpha statistics causes near-equivalent items to be combined as though they were statistically independent [34]. Third, error is assumed to be constant across the distribution of measurement whereas it is likely to vary (and so be less-suited, or demand larger samples, for some studies targeted at either end of the continuum). Finally, the procedures and justification for combining subscales into a single global score are not widely understood and so much research use multifactorial instruments as though they were unidimensional, despite having evidence to the contrary [35].

Rasch analysis has been proposed as a robust measurement paradigm [36]. Developed in education sciences as a means for measuring aptitude, Rasch analysis proposes that the likelihood of a person agreeing with a questionnaire statement is related to its ‘difficulty’. That is, some items are easier to agree with than others, and do not equally convey the same information about quality. Thus, a positive response to a statement that very few other people agree with would likely suggest that the respondent occupies a higher position on the latent continuum (whereas a CTT scale pays no regard to item difficulty). Where a consistent hierarchical structure exists within the questionnaire (a probabilistic form of the Guttman pattern) Rasch analysis forms estimates of each respondent’s location on the latent scale [34]. In the event that an instrument can demonstrate it satisfies a series of assumptions (see below), the resulting measure is assured interval-level properties suited for parametric hypothesis testing in research [34]. Furthermore, Rasch analysis permits detailed investigation of local dependence problems and the distribution of error across the continuum.

This paper aims to establish whether the PCQ-S conforms to Rasch assumptions and to provide a mechanism for researchers to convert raw scores to interval-level Rasch scores.

## Methods

This study uses cross-sectional data collected as part of the Umeå ageing and health research program in Sweden (U-Age) [37]. The PCQ-S was administered through a self-administered questionnaire distributed to nursing home staff between November 2013 and September 2014. Further detail is as follows.

### Participants

The U-Age data collection consisted of nationwide randomly selected nursing homes and their residents. A total of 60 (of 290) Swedish municipalities were randomly selected for the project and of those 35 agreed to participate. Within these municipalities, nursing home managers were contacted by telephone and 188 of 202 invited nursing homes agreed to participate. No further attempts were made to approach non-participating municipalities or units to find their reasons for not participating. This study was based on data from 172 nursing homes, since 16 did not return data although they had agreed to participate. The sample comprised staff working within 548 units. Further sampling details are available elsewhere [37].

### Analysis

Rasch analysis was implemented through a partial credit model [38] suitable for polytomous items, within RUMM2030 software. The objective of a Rasch analysis is to construct a scale from individual items and test its suitability for interval level measurement. Scale construction is possible by using a logistic function to relate a person’s probability of answering an item using a given response category to their underlying position on the latent continuum. Scores are thus measured in logits. RUMM2030 enables inspection of key assumptions to be satisfied [34], specifically:

1. Overall fit: A χ^2^ statistic assesses the overall fit to the Rasch model (against the null hypothesis of perfect fit). In addition, the distribution of standardised residuals for both persons and items should have a standard deviation no larger than 1.4.
2. Item and person fit: Individual person and item residuals should be as close to zero as possible. Residuals in excess of ±2.5 are considered potentially problematic. At the item level, large negative residuals indicate redundancy (akin to very high item-total correlations in CTT). These are evaluated within RUMM2030 using an F-test.
3. Threshold ordering: Each response category should be in the anticipated order and each should have the greatest likelihood of being chosen for some part of the latent continuum.
4. Local dependence: Items should not be correlated beyond that associated with the construct under measurement. Within RUMM2030, this is evaluated through the item residual correlation matrix, with those correlations larger than the mean + 0.20 regarded as problematic.
5. Unidimensionality: Rasch models require that only one construct is being measured. RUMM2030 conducts a principal components analysis of residuals, with negatively / positively loading items then separately used to estimate each respondent’s location on the logit scale. Paired t-tests then estimate the significance of these differences. The proportion of t-tests reaching significance should not exceed 5%.
6. Reliability: The internal consistency is estimated using the Person Separation Index and Cronbach alpha. A PSI in excess of 0.70 is generally viewed as a minimum for research purposes.
7. Differential Item Functioning: In this study, we use a set of general personal and job-related characteristics (gender, age, qualification, and type of care setting) to evaluate whether some groups of respondents have a different likelihood of answering an item / category despite being located at the same point on the latent continuum. DIF may be uniform (a constant differential across the scale) or non-uniform (where differential varies across the scale) and is evaluated through an ANOVA.

Against a null hypothesis of perfect fit, the Rasch tests are known to be over-powered for large n (e.g. *n* > 400); that is, even acceptable levels of deviation from the Rasch assumptions are found to be statistically significant. Because a large sample was achieved in this study (described below), two 10% random subsamples were drawn without replacement from the full dataset, following the approach of Gibbons et al. [39]. Statistical tests confirmed that there were no significant differences in the characteristics of the two samples. Rasch analysis was first conducted on subsample 1 (the ‘evaluation sample’), which was then reapplied in subsample 2 (the ‘validation sample’); with the stability of fit indicators assessed in both applications. For the purpose of describing the final item locations, standard errors, and a nomogram for converting raw to logit scores, the model was re-estimated in the full sample.

The PCQ-S has been found in previous testing to be best represented by a three-factor structure (see above). Three separate scales were therefore constructed and separately tested. To form a summary scale from the three factors, ‘subtests’ were formed within RUMM2030 (see [38] for a similar example of this process). Under subtests, the items within each subscale are combined and entered as ‘meta-items’ within the Rasch model. Since subtests parcel-out dependency between items, this necessarily reduces internal consistency estimates. In the event that a single summary score formed of these subtests satisfies the Rasch fit assumptions, then this ‘higher order’ Rasch scale is able to resolve the problems caused by dependency between subsets of items. 

## Results

Of 6902 questionnaires distributed to staff, 4831 were returned representing a 70% response rate. The broad sample characteristics are presented in Table 2. Over 80% of the sample were registered nurses with others representing different grades of non-registered practitioners. Approximately a third were based in group living environments with the bulk of the sample drawn from nursing homes. The size of participating units ranged from 7 to 128 beds and included both general as well as special care units for dementia. As noted above, Rasch analysis was performed in ‘evaluation’ and ‘validation’ subsamples randomly drawn from this dataset.

**Table 2:** Demographics of the study group

		Number	Percent
Gender (missing = 35)	Female	3414	95.4
	Male	166	4.6
Age (years) (missing = 133)	≤30	466	12.9
	31–40	622	17.2
	41–50	1061	29.3
	51–60	1103	30.5
	> 60	363	10.0
Registration/qualification status (missing = 67)	Staff nurse	2921	82.3
	Care assistant	470	13.2
	Other	73	2.0
	No formal education	84	2.4
Care setting type (missing = 55)	Group living	1153	32.4
	Nursing home	2187	61.4
	Other	220	6.3
Care setting size (no. of beds) (missing = 103)	≤10	1551	43.3
	11–20	1497	41.8
	> 20	535	14.9

### Subscale 1: a climate of safety

Initial Rasch analysis of items 1–5 indicated a poor fit (*p* < 0.0001, see Table 3). There was evidence of disordered thresholds in four items, which were resolved by rescoring each by combining responses falling in the second and third categories. There appeared to be two additional causes of misfit. First, there were large residuals for items 1 and 5 and, second, evidence of local dependency between item 1 and 2 . Item 1 had a large negative residual indicating over-discrimination and redundancy and was the only item with a statistically significant F-statistic. Upon its removal, the Rasch assumptions were met (χ^2^(20) = 26.112, *p* = 0.162) with items fitting appropriately, albeit with a slightly larger than expected residual standard deviation. These four items formed a unidimensional subscale, with under 3% of paired t-tests reaching significance. The thresholds for each item category are shown in accompanying category characteristics curves in Additional file 1. Robustness of fit was tested by re-examining these four items in the validation sample, with similar results being achieved. No evidence of DIF was identified for gender, age group, qualification status, type of care setting or its size (full results from RUMM2030 for DIF analyses are provided as Additional file 2).

**Table 3:** Summary fit statistics (scales)

Analysis	# items	Chi square		Item residual		Person residual		Reliability		Unidimensional (% sig t-tests)
		Value	*p*	Mean	s.d.	Mean	s.d.	PSI	α^a^	
Subscale 1: A Climate of Safety										
Initial	5	52.99	< 0.001	0.068	2.483	−0.378	0.997	0.70	0.82	8.33%
Final	4	26.11	0.162	0.193	1.541	−0.408	0.994	0.60	0.76	2.90%
Validation	4	23.39	0.104	0.260	1.572	−0.307	0.920	0.52	0.78	2.32%
Subscale 2: A Climate of Everydayness										
Initial	5	41.62	0.020	−0.303	2.041	−0.466	1.094	0.82	0.86	6.94%
Final	4	21.01	0.396	0.099	1.234	−0.396	0.998	0.76	0.81	3.18%
Validation	4	30.07	0.068	0.288	1.135	−0.404	1.008	0.74	0.81	2.04%
Subscale 3: A Climate of Community										
Initial/Final	4	26.23	0.158	−0.139	1.260	−0.316	0.757	0.59	0.84	3.47%
Validation	4	25.61	0.060	0.027	1.168	−0.272	0.738	0.55	0.83	1.73%
Summary Scale										
Initial	12	77.80	0.061	−0.155	1.540	−0.345	1.127	0.85	0.90	9.17%
Subtests	12	17.44	0.293	−0.302	0.728	−0.350	0.947	0.76	0.81	2.66%
Validation	12	21.56	0.120	−0.358	1.224	−0.408	0.880	0.71	0.79	1.61%
Ideal values	–	0	< 0.01	0	< 1.4	0	< 1.4	> 0.70	> 0.70	< 5%

*sd* standard deviation

^a^Cronbach alpha is only available for participants providing complete responses (and hence is for a smaller sample than the PSI)

### Subscale 2: a climate of everydayness

Analysis of items 6–10 indicated some misfit to the Rasch model as evidenced by a borderline significant χ^2^ value and a large item residual standard deviation. Items 7,8 and 10 all required rescoring in the same form as for items in subscale 1 to resolve disordered thresholds. A potential source of misfit was due to local dependence between items 8 and 9 indicating shared variation beyond the latent trait under measurement. Item 8 was on the threshold of significant misfit, and so was removed. The reduced scale had a non-significant χ^2^ value, was unidimensional and free from local dependency. Applying the same model to the validation sample gave satisfactory results. As for subscale 1, no evidence of (uniform or non-uniform) DIF was identified for any variables tested.

### Subscale 3: a climate of community

Analysis of items 11–14 revealed good fit to the Rasch model including a non-significant χ^2^ value, with satisfactory distribution of residuals and was unidimensional. All thresholds were ordered without need for rescoring. Analysis of residual correlations indicated some evidence of local dependence between items 13 and 14 (a place where it is easy for patients to talk to staff/a place where patients have someone to talk to). The residual correlation was 0.29 greater than the mean correlation in the matrix. However, removing or combining the items caused other fit difficulties and so the two separate items were kept. No evidence of DIF was identified.

### Summary scale

The 12 items forming the three subscales (referred to hereafter as the PCQ-S-R, denoting the Rasch version) were then combined in a single model, showing good fit to Rasch assumptions except for its (anticipated) multidimensionality. The three subscales were then formed as ‘testlets’ within RUMM2030 and re-estimated, showing generally good fit, as evidenced by non-significant χ^2^ value and non-significant test of unidimensionality once subscales were accounted for. The person separation index for the summary scale was 0.776, which was above the required minimum levels for research purposes (=0.70). Re-estimation within the validation sample supported these findings (see Table 3). Table 4 presents the item level results for the final model.

**Table 4:** Summary fit statistics (items)

	Scoring	Location	SE	Fit residual	F-statistic	Prob
*Subscale 1: A Climate of Safety*						
2. A place where I feel acknowledged as a person	0,1,1,2,3,4	0.557	0.079	− 0.802	1.185	0.316
3. A place where I feel I can be myself	0,1,1,2,3,4	0.423	0.080	− 1.274	1.104	0.358
4. A place where the patients are in safe hands	0,1,1,2,3,4	−0.539	0.088	0.738	1.583	0.156
5. A place where the staff use a language that the patients can understand	0,1,2,3,4,5	−0.441	0.083	2.112	1.336	0.249
*Subscale 2: A Climate of Everydayness*						
6. A place which feels homely even though it is in an institution	0,1,2,3,4,5	−1.280	0.088	−0.566	2.171	0.057
7. A place where there is something nice to look at	0,1,1,2,3,4	0.367	0.077	−0.036	0.791	0.557
9. A place where it is possible to get unpleasant thoughts out of your head	0,1,2,3,4,5	0.603	0.067	−0.878	1.477	0.197
10. A place which is nice and clean	0,1,1,2,3,4	0.311	0.077	1.875	0.699	0.624
*Subscale 3: A Climate of Community*						
11. A place where it is easy for the patients to keep in contact with their loved ones	0,1,2,3,4,5	0.284	0.093	1.678	0.669	0.647
12. A place where it is easy for the patients to receive visitors	0,1,2,3,4,5	−0.346	0.105	−0.767	1.223	0.299
13. A place where it is easy for the patients to talk to the staff	0,1,2,3,4,5	−0.692	0.100	−1.156	2.126	0.063
14. A place where the patients have someone to talk to if they so wish	0,1,2,3,4,5	0.753	0.087	−0.312	1.502	0.190
*Summary scale*						
Subscale 1	–	−0.150	0.031	0.431	1.527	0.181
Subscale 2	–	0.268	0.027	−1.123	1.455	0.204
Subscale 3	–	−0.118	0.030	−0.689	1.158	0.330

**Fig. 1:**
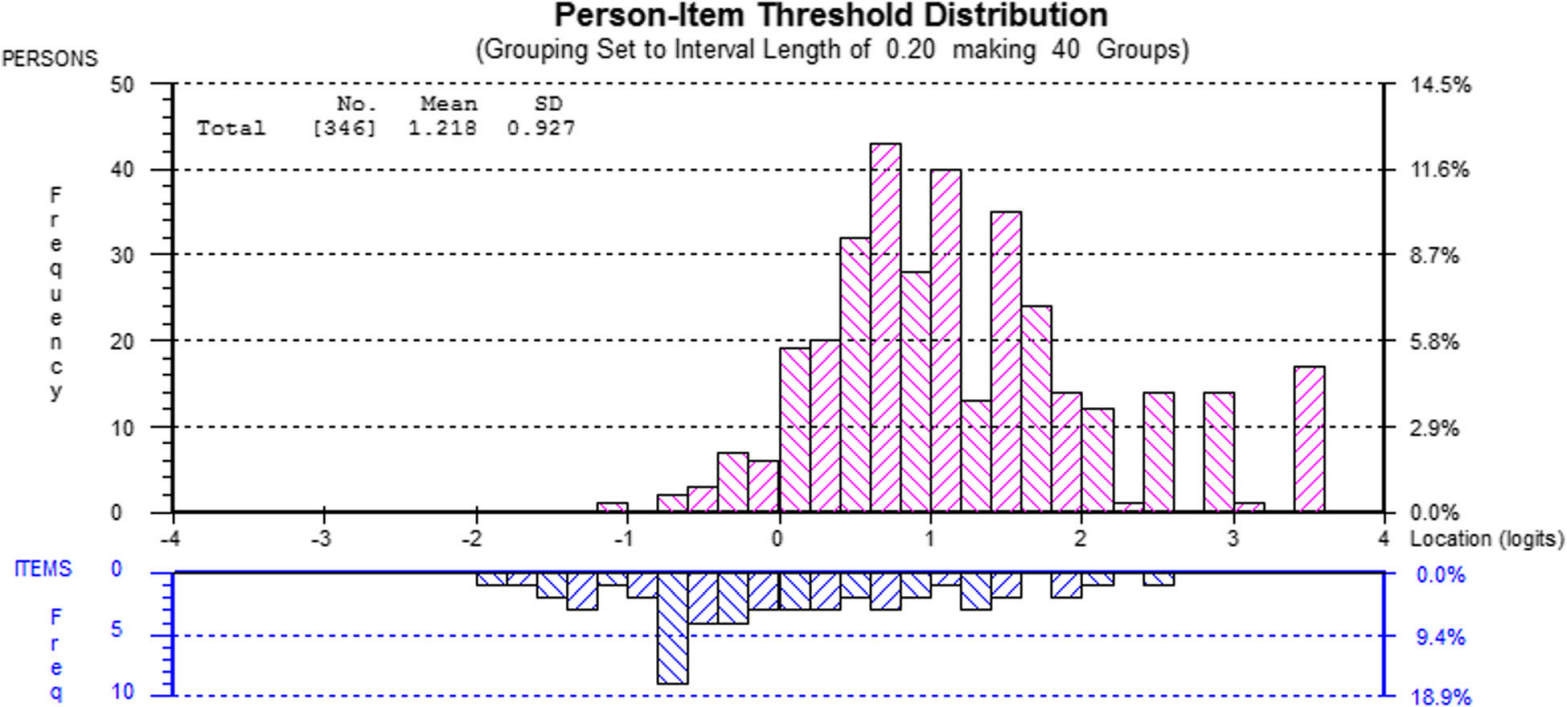
Person-Item Threshold Distribution

The PCQ-S-R was then estimated for the whole sample. Figure 1 presents the distribution of respondents and item thresholds and indicates targeting problems. With item thresholds anchored with a mean of zero logits (lower panel) the mean of the person distribution was 1.10 logits (standard deviation of 0.86), with 7.6% of respondents at extreme values. A more efficient scale would have questions that were less often affirmed in the sample. Finally, Table 5 presents a nomogram enabling researchers to convert ordinal raw scores to metric logit scores (and rescaled logit scores matching the range of the raw score).

**Table 5:** Nomogram converting PCQ-S 12 item raw score to logit score and rescaled logit score

Raw score	Logit score	Rescaled logit score	Raw score	Logit score	Rescaled logit score	Raw score	Logit score	Rescaled logit score
0	−1.796	0.00	21	−0.516	15.58	41	0.25	24.91
1	−1.348	5.45	22	−0.498	15.80	42	0.31	25.64
2	−1.132	8.08	23	−0.48	16.02	43	0.371	26.38
3	−1.029	9.34	24	−0.46	16.27	44	0.434	27.15
4	−0.96	10.18	25	−0.44	16.51	45	0.499	27.94
5	−0.907	10.82	26	−0.417	16.79	46	0.567	28.77
6	−0.864	11.35	27	−0.393	17.08	47	0.637	29.62
7	−0.827	11.80	28	−0.367	17.40	48	0.712	30.54
8	−0.795	12.19	29	−0.337	17.76	49	0.791	31.50
9	−0.764	12.56	30	−0.304	18.17	50	0.875	32.52
10	−0.738	12.88	31	−0.266	18.63	51	0.966	33.63
11	−0.712	13.20	32	−0.224	19.14	52	1.066	34.85
12	−0.688	13.49	33	−0.178	19.70	53	1.177	36.20
13	−0.666	13.76	34	−0.129	20.30	54	1.302	37.72
14	−0.647	13.99	35	−0.079	20.91	55	1.445	39.46
15	−0.628	14.22	36	−0.027	21.54	56	1.615	41.53
16	−0.607	14.48	37	0.026	22.18	57	1.824	44.07
17	−0.59	14.68	38	0.08	22.84	58	2.098	47.41
18	−0.57	14.93	39	0.136	23.52	59	2.511	52.44
19	−0.554	15.12	40	0.192	24.20	60	3.132	60.00
20	−0.536	15.34						

## Discussion

Amid clarion calls to improve measurement in person-centred care [40], this paper sought to bolster the quality and rigour of one such instrument through application of Rasch analysis to the PCQ-S. The PCQ-S is one of the most widely used questionnaire-based measures of person-centredness in dementia research, being translated into multiple languages and growing evidence of its measurement properties [17]. However, all current work has used classical test theory, leaving the PCQ-S open to concerns over how Likert-type items are simply summed to form the measure. This new research found that a 12-item version, labelled the PCQ-S-R, broadly satisfied the assumptions of the Rasch model by forming subscales from the three separate factors. A notable strength of the analysis is the large sample size on which it is drawn. By using the nomogram, researchers using the PCQ-S-R can be satisfied that any subsequent analysis would be of interval-level scores.

A further advance of the PCQ-S-R is that a single score can be used to accurately represent respondents’ perceptions of person-centredness, rather than relying on three distinct but correlated subscales. Unless a particular dimension is the subject of attention, combining the three scales into a global measure of person-centredness would be a researcher’s preference. Although it is commonplace to sum subscale scores under CTT into a global score, few applications have formally assessed (e.g. through bifactor modelling) how appropriate this would be for the construct of interest. Not all subscales comprise sufficient common variance to justify aggregation [41]. However, the PCQ-S-R, formed of three subscales, passes the Rasch assumptions.

An important feature of the PCQ-S lies in its spread of content across themes that resonate strongly with person-centred literature. However, the response patterns for two items did not accord with Rasch expectations, and so were removed in the PCQ-S-R. Item 1 (‘a place where I feel welcome’) was removed, which might be of some concern given this has been identified as an important aspect of service user and carer experience, at least in hospital settings [42]. Arguably, the notion of ‘feeling welcome’ is pertinent in joining new environments that is not one’s own, and may be less suited to long-term residential settings where some will have been resident for many months and years. It is therefore plausible that other items more accurately capture the essence of what was intended, as is indicated by the Rasch analysis. Similarly, the Rasch analysis found evidence of local dependency between item 8 (‘a place that is quiet and peaceful’) and item 9 (‘a place to get unpleasant thoughts out of your head’). Presumably respondents considered that these were tautological, and therefore the removal of item 8 would not be a considerable loss to the content validity of the scale. Additional research interviews with respondents to the scale would be useful to explore these redundancy issues further.

Rasch analysis has the added advantage of investigating the efficiency of a scale. The results suggest that the PCQ-S suffers from mistargeting. Many of the Likert thresholds at the lower end of the spectrum contribute little information, since so few individuals report care quality that is so poor. By contrast, at the higher quality end of the spectrum, too few thresholds mean that it is challenging to discriminate between respondents’ perceptions. The scale is therefore less-suited for monitoring change in services where person-centredness quality is already strong. Ideally, the PCQ would contain more items that, to be endorsed, would require even higher standards of person-centredness. Targeted qualitative work with those already perceiving that services are of a high quality could help to identify more ‘difficult’ standards for inclusion in the PCQ. In the future, further items could potentially form an item bank for use within computer adaptive testing (whereby questions are tailored during the test depending on earlier responses, to identify more accurate estimates of the phenomenon under measurement.)

The analysis is not without its limitations. First, the analysis relates to the Swedish language version of the PCQ-S, and it cannot be assumed that the same conclusions would apply to other versions. The challenges in creating semantically and culturally equivalent scales are well known [43], and formal analysis would require parallel application using other language versions. Second, the sample is restricted to residential settings and there can be no guarantees that the same findings would have been achieved from hospital-based respondents. That said, there is some reassurance from the absence of any Differential Item Functioning between the nursing homes and other settings within the sample. Finally, it is worth recalling that the PCQ-S relies on staff reports and these may differ from independent ratings of person-centredness in any given facility. Arguably, on average, staff will report more positive views of person-centredness within their facility than outside observers. Ideally these questions require improvement to clarify their distinction and this should be the focus of future research.

## Conclusions

The PCQ-S-R is a 12-item scale that can be used to appraise the person-centredness in dementia care settings. The research represents a significant advance since the questionnaire can now be said to have been examined against the rigorous assumptions of the Rasch model, and can be more confidently analysed using parametric statistical procedures. Furthermore, the research offers a means for correctly calculating a single global score rather than three separate subscales. Yet some improvements are still required. Specifically, the scale is mistargeted, meaning that it may not be sensitive to change at higher levels of person-centred quality, and further research could explore and refine two items that may still be locally dependent.

## Supplementary information

**Additional file 1.** Category Characteristics Curves.

**Additional file 2.** Differential Item Functioning.

## Data Availability

The datasets used and/or analysed during the current study are available on reasonable request.

## Abbreviations

CTT: Classical Test Theory
DIF: Differential Item Functioning
PCQ-S: Person-centred Climate Questionnaire – Staff
PCQ-S-R: Person-centred Climate Questionnaire – Staff – Rasch version
PSI: Person Separation Index
