# Supplementary figures and images for: A Rasch analysis of the Person-Centred Climate Questionnaire – staff version
Source: BMC Health Serv Res. 2019 Dec 26;19:996. doi: 10.1186/s12913-019-4803-9 (PMC6933628; doi:10.1186/s12913-019-4803-9)

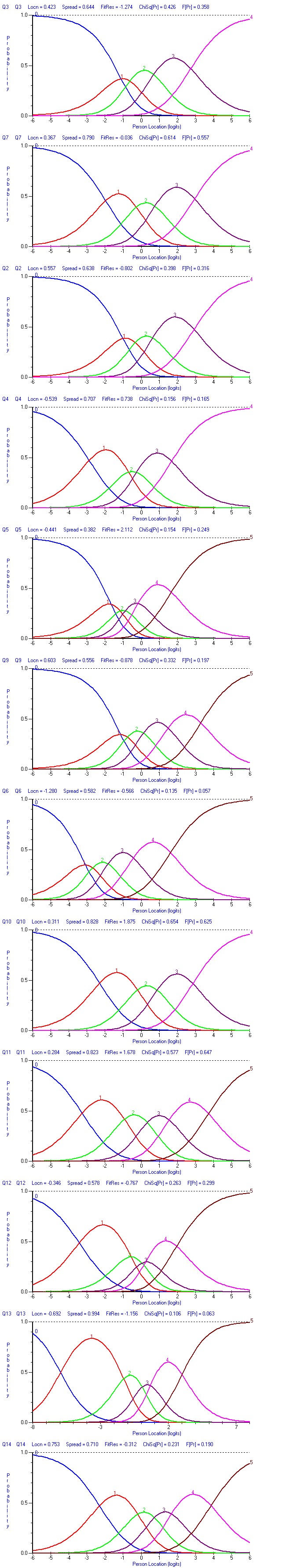

Supplement: Supplementary file 1 — Additional file 1. Category Characteristics Curves. [file 12913_2019_4803_MOESM1_ESM.jpg]
